# Radiotherapy combined with anlotinib for recurrent high-grade soft tissue sarcoma of the scalp: a case report

**DOI:** 10.3389/fonc.2026.1941568

**Published:** 2026-10-14

**Authors:** Bingxuan Li, Jinling Ren, Xiaoxia Song, Xiaogai Qi

**Affiliations:** 1 Department of Oncology, Shangdong Provincial Key Medical and Health Discipline, Qingdao Central Hospital, University of Health and Rehabilitation Sciences, Qingdao, Shandong, China; 2 Emergency Center, Qingdao Central Hospital, University of Health and Rehabilitation Sciences, Qingdao, Shandong, China

**Keywords:** anlotinib, case report, complete response, radiotherapy, soft tissue sarcoma

## Abstract

**Purpose:**

Soft tissue sarcoma (STS) is a rare but aggressive mesenchymal malignancy that accounts for less than 1% of all adult cancers. Head and neck soft tissue sarcomas (HNSTS) are infrequent malignant tumors arising from mesenchymal and neuroectodermal tissues in the head and neck region with a poor prognosis, and recurrence is predominantly local. There are few targeted management guidelines.

**Case description:**

We report the case of a 77-year-old woman with postoperative recurrence of HNSTS. The patient was diagnosed with high-grade soft tissue sarcoma of the scalp in February 2025 and treated with surgery. In June 2025, the patient was diagnosed with recurrence, and was subsequently treated with combination therapy consisting of the anti-angiogenic inhibitor anlotinib and radiotherapy. In December 2025, after nine cycles, the patient showed a complete response (CR) to therapy.

**Conclusion:**

This indicates that radiotherapy combined with anti-angiogenic targeted therapy may produce synergistic effects in patients with HNSTS and provide directions for future clinical decision-making.

## Introduction

Head and neck soft tissue sarcomas (HNSTS) are rare soft tissue sarcomas (STS) with a dismal prognosis, accounting for less than 1% of all head and neck cancers (1, 2). Its pathogenesis remains unclear, though it has been associated with multiple risk factors including radiotherapy, chronic lymphedema, various chemical agents, and immunodeficiency (3). HNSTS mainly occur in the neck, the face and forehead, the maxilla, and the paranasal sinuses and pharynx (4). Unlike STS at other sites, patients with HNSTS often die as a result of local recurrence. Currently, extensive surgical resection and radiotherapy play a crucial role in the prognosis of head and neck STS (5).

Various external beam radiotherapy techniques have been applied in postoperative radiotherapy for HNSTS, showing significant efficacy in improving local control (LC) rates and overall survival (OS) (6). In recent years, with the gradual emergence of targeted therapies, an increasing number of targeted drugs have also demonstrated good clinical efficacy in patients with certain histological types of advanced STS (7). The combination of anti-angiogenic agents and chemotherapy has emerged as the new standard of first-line treatment for advanced STS (8). Anlotinib is a novel multitarget tyrosine kinase inhibitor that targets angiogenesis-related kinases such as vascular endothelial growth factor receptor (VEGFR)1/2/3, fibroblast growth factor receptors (FGFR)1/2/3, and other tumor-associated kinases such as c-Kit and RET (9, 10). A recent study has shown an expanding role for anlotinib-based combination strategies in solid tumors (11). Previously, the efficacy of anlotinib in the treatment of advanced STS has been demonstrated in several studies (12, 13). However, radiotherapy combined with anlotinib has not been reported for the treatment of patients with recurrent high-grade soft tissue sarcoma of the scalp till now. Here, we report a case of a patient with postoperative recurrence of a high-grade soft tissue sarcoma of the scalp. Complete response (CR) was achieved through combined local and systemic therapy, including brain radiation and anti-angiogenic targeted therapy.

## Case description

A 77-year-old female patient was admitted to our hospital in June 2025 with postoperative recurrence of high-grade soft tissue sarcoma of the scalp. The patient’s parents were deceased; her father died in an accident and her mother died of a heart attack. She had two siblings: one brother and one sister, both of whom are alive and have no history of cancer. There is no history of mental illness, nor has the patient ever smoked or consumed alcohol. The patient underwent a tumor resection in the forehead at another hospital on 26 February 2025. The patient had a 20-year history of coronary heart disease with the condition well controlled on oral medication and underwent surgery for a meningioma in 2006, followed by postoperative radiation therapy; the specific dose was unknown. There was no history of other surgeries. Postoperative pathological examination (PE) suggested a spindle cell tumor (frontal tumor) with extensive necrosis, marked cellular atypia, and prominent mitotic figures. Immunohistochemistry stains were as follows: CK (−), EMA (−), PR (−), SSTR2 (−), S100 (−), GFAP (−), Ki-67 (+, 70%), Olig2 (−), CD34 (−), CD99 (−), Desmin (−), SMA (−), BCL2 (−), HMB45 (−), Melan-A (−), D2-40 (−), CD4 (−), ALK (4A4) (−), CD21 (−), CD30 (−), CD68 (−), CD163 (−). Based on the immunohistochemical results, the diagnosis was high-grade sarcoma. Further subtyping was difficult. Postoperative recovery was satisfactory. In June 2025, the patient’s scalp lesion recurred on the left forehead; it measured approximately 2 cm × 2 cm, was firm and fixed, and appeared purplish-red. Positron emission tomography/computed tomography (PET/CT) scanning disclosed post-surgical findings following resection of a subcutaneous epidural sarcoma: two hypermetabolic nodules in the left-sided scalp region of the forehead and a hypermetabolic nodule in the left frontal meningeal region; sarcoma metastasis was suspected (**Figure 1**). The patient was classified as high-grade scalp sarcoma, T4N0M1, and brain parenchymal metastasis.

**Figure 1:**
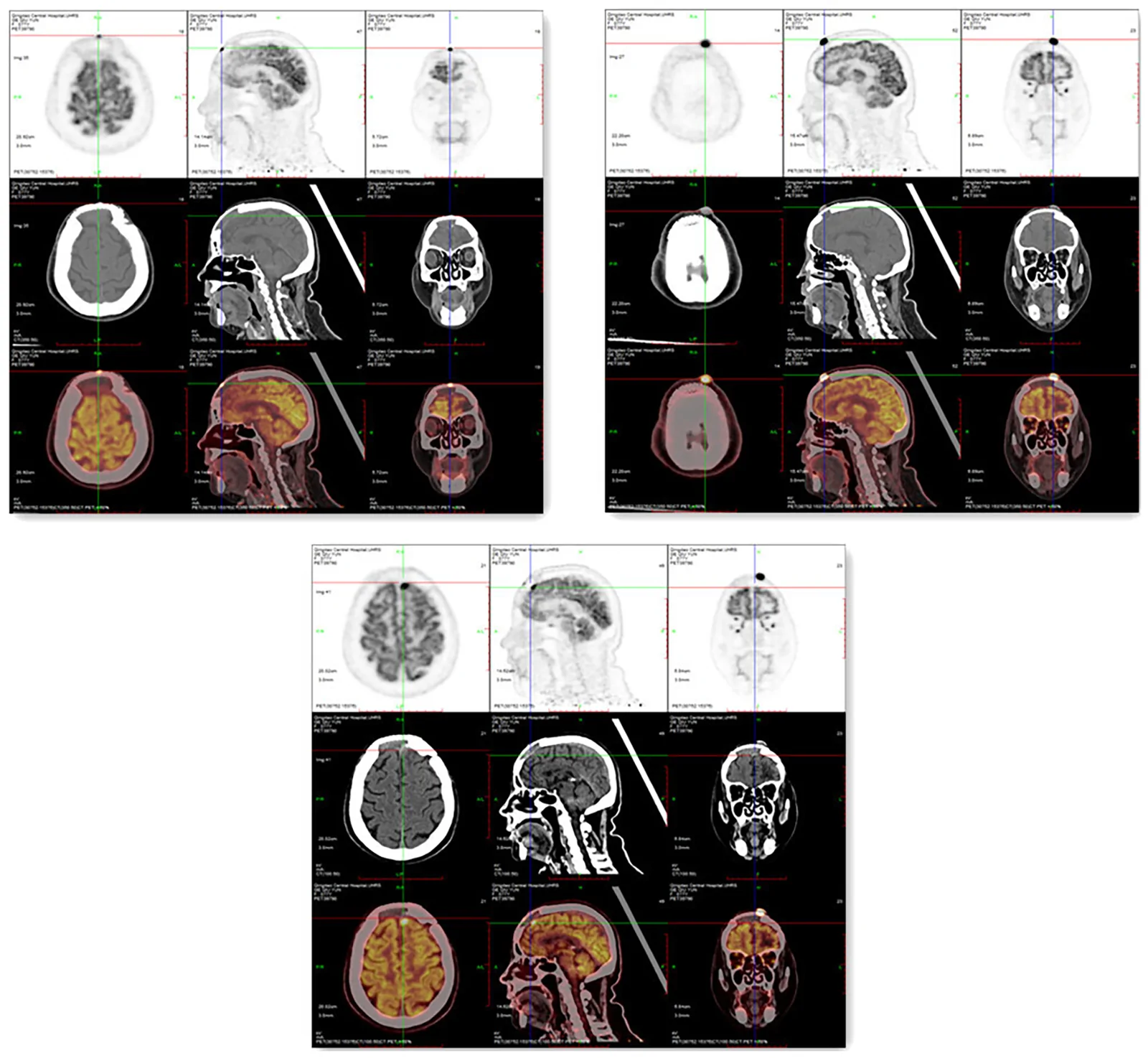
18F-FDG-PET/CT suggested two hypermetabolic lesions on the left frontal scalp **(A, B)**; metastatic hypermetabolic lesions in the left frontal lobe **(C)**.

From 12 June 2025, the patient received simultaneous integrated boost helical tomotherapy for recurrent scalp sarcoma lesions and the tumor bed followed by targeted therapy with synchronous oral administration of anlotinib. The prescribed doses were 66 Gy in 30 fractions of 2.2 Gy per fraction for the planning gross tumor volume of the primary lesion (PGTVp), and 60 Gy in 30 fractions of 2 Gy per fraction for the planning gross tumor volume of the tumor bed (PGTVtb). Anlotinib was prescribed as per the standards (12 mg daily orally for 14 consecutive days, repeated every 3 weeks, 21 days as a cycle). The specific irradiation field is shown in **Figure 2**. On 22 July 2025, the radiotherapy ended; the patient tolerated the radiotherapy and other interventions well. Anlotinib as maintenance therapy, was continued for a long time. During this period, there was no adverse event related to anlotinib, such as hypertension or proteinuria. A follow-up brain magnetic resonance imaging (MRI) performed in August 2025 showed that compared with that before radiation, both the number and size of the patient’s scalp and intracranial metastases had decreased (**Figure 3**). In October 2025, four months after radiotherapy, the brain MRI examination showed that the scalp and intracranial metastases were gradually controlled and evaluated as a partial response (PR) according to the Response Evaluation Criteria in Solid Tumors (RECIST 1.1) (**Figure 3**). At the follow-up on 16 December 2025, an MRI review showed that the scalp lesions had completely disappeared, and no new lesions had appeared with the treatment efficacy being evaluated as a CR (**Figure 3**). Anlotinib was prescribed continuously until February 2026, after which treatment was discontinued due to a pulmonary infection and heart failure. As of May 2026, the patient had demonstrated excellent tumor control. Unfortunately, the patient ultimately died from a lung infection complicated by heart failure on 4 May 2026, with an OS of 11 months from the diagnosis of a recurrence and a suspected intracranial lesion in June 2025.

**Figure 2:**
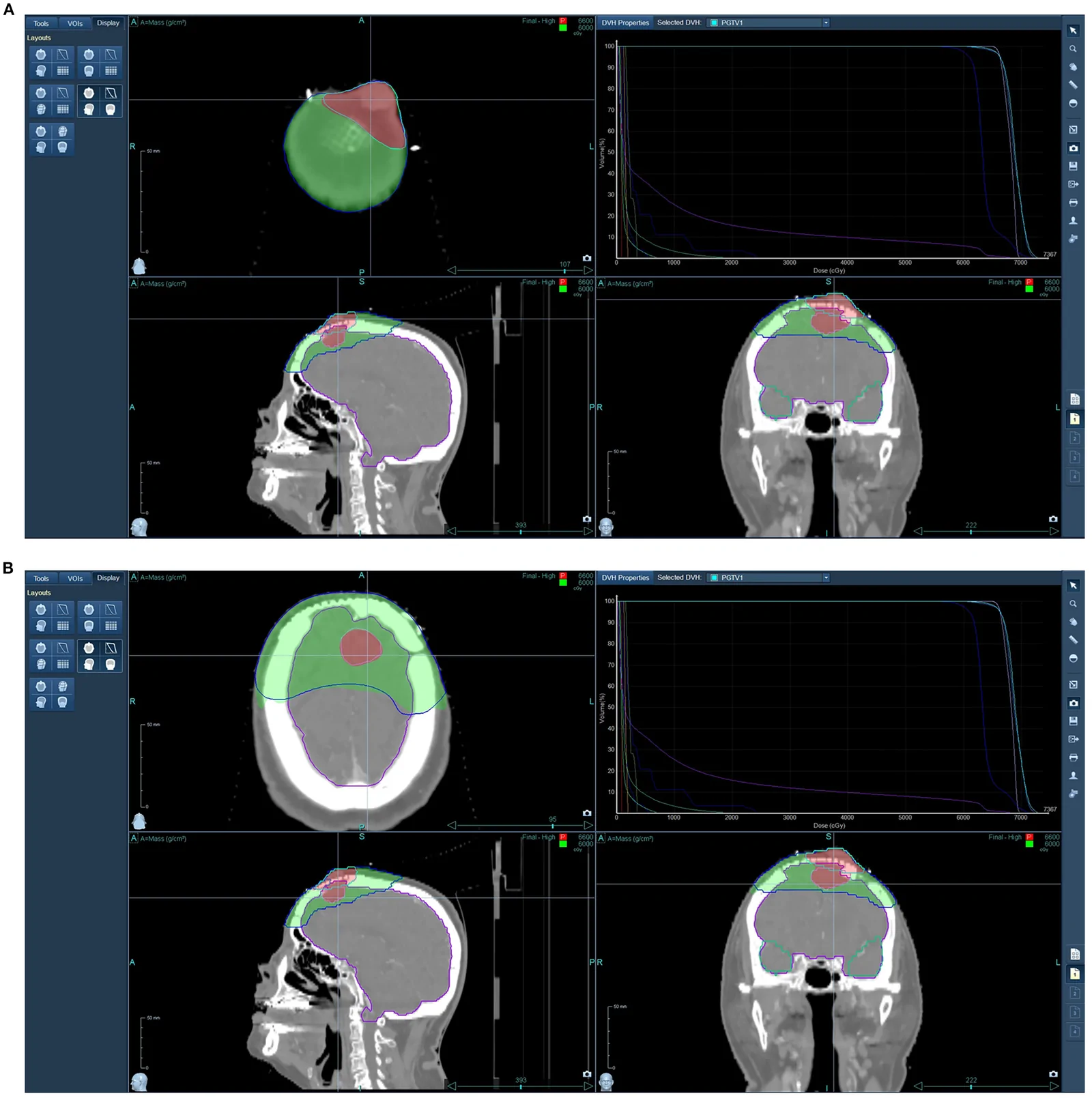
Illustration of the patient’s radiation treatment planning and dose distribution. Individualized brain irradiation utilizing the simultaneous integrated boost helical tomotherapy for recurrent scalp **(A)**, left frontal lobe metastatic lesions **(B)**, and tumor bed. The red and green isodose color-wash indicates doses of 66 Gy (PGTVp) and 60 Gy (PGTVtb), respectively.

**Figure 3:**
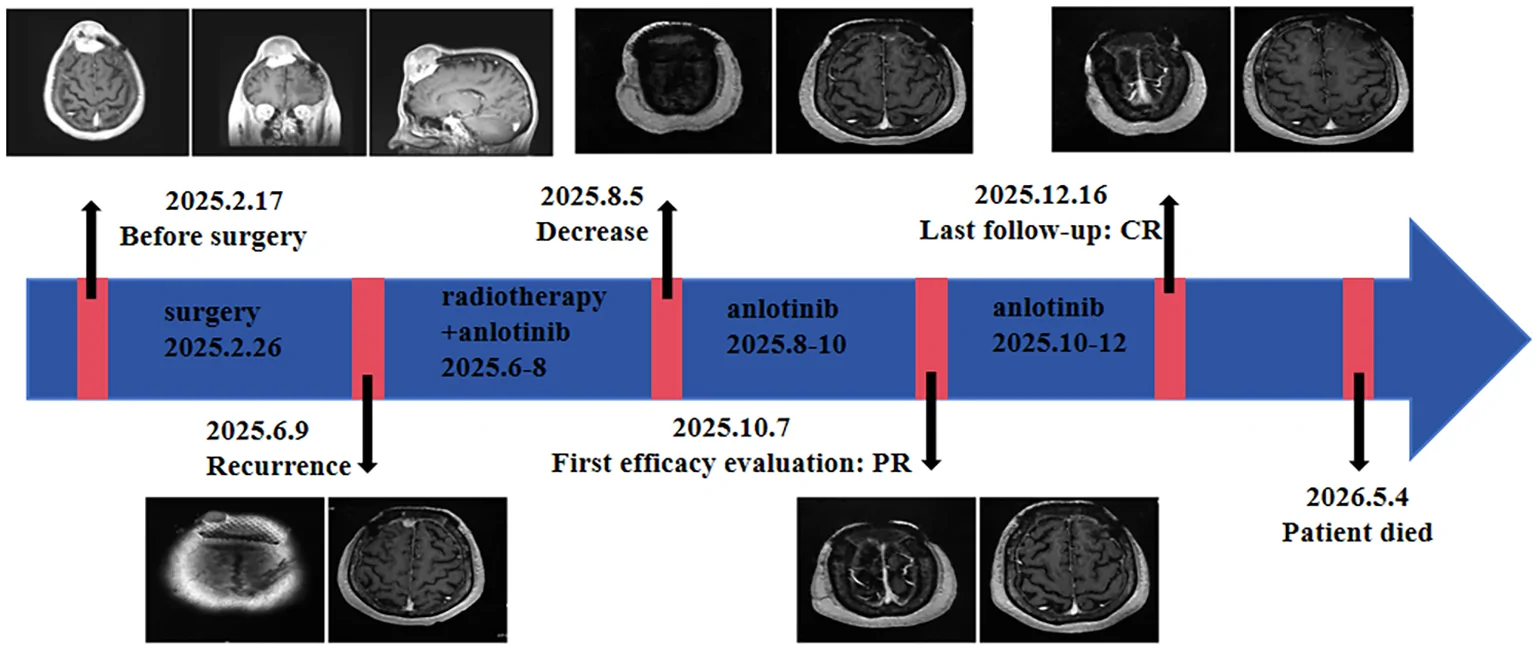
Flow chart of the whole treatment. Brain MRIs of the patient were displayed. **(A)** MRI of the head upon patient’s admission in February 2025 displays space-occupying lesions in the frontal region (prior to surgery). **(B)** The MRI of the head from June 2025 shows a postoperative recurrence (prior to radiation therapy). **(C)** Follow-up MRI of the head after radiation therapy and anlotinib in August 2025 demonstrates that, compared with that before radiation, both the number and size of the patient’s scalp and intracranial metastases had decreased. **(D)** Follow-up MRI of the head in October 2025 shows that four months after radiotherapy, the scalp and intracranial metastases were gradually controlled and evaluated as PR. **(E)** The follow-up MRI of the head in December 2025 shows that the scalp lesions had completely disappeared, and no new lesions had appeared with the treatment efficacy being evaluated as a CR.

## Discussion

HNSTS are rare aggressive tumors, constituting a very heterogeneous group (14, 15). The prognosis for HNSTS in adults is so poor that the 5-year overall survival rate is approximately 60% (1). Recurrence is predominantly local. The most common histological subtypes include malignant fibrous histiocytoma (MFH), fibrosarcoma, angiosarcoma, malignant peripheral nerve sheath tumors, and unclassified/undifferentiated sarcomas (16, 17). Due to its rarity, there are currently no specific treatment guidelines for this disease. The treatment of HNSTS is usually based on the standard protocols used for soft tissue sarcomas of the extremities (6). For high-grade lesions or cases involving microscopic residual disease, surgery combined with radiotherapy is the standard treatment regimen (18).

With the gradual rise of targeted therapy, the combination of anti-angiogenic agents and existing chemotherapy regimens is increasingly becoming a crucial treatment strategy for STS. Anti-angiogenic drugs can inhibit abnormal angiogenesis in STS and restore vascular normality (19). Furthermore, anti-angiogenic drugs have low toxicity and high safety. They are unlikely to cause more adverse reactions while ensuring efficacy, making them particularly suitable as a maintenance therapy for patients with advanced STS (20). Anlotinib is a novel oral small-molecule multi-target tyrosine kinase inhibitor (TKI). It exerts anti-tumor effects primarily by inhibiting the vascular endothelial growth factor (VEGF) receptors (1/2/3) and other major tyrosine kinase receptors, such as FGFR1-4, PDGFR a/b, c-Kit, and FLT3 (21).

Anlotinib has currently been approved for the treatment of advanced soft tissue sarcoma and lung cancer, and has demonstrated significant efficacy in patients with advanced hepatocellular carcinoma (8, 22, 23). A previous study has provided evidence that anlotinib-based combinations can improve outcomes in another solid-tumor setting (24). First-line anlotinib combined with SBRT for the treatment of lung sarcoma results in significant tumor response, achieving partial remission (PR) (25). Meanwhile, whole brain radiotherapy (WBRT) plus anlotinib in non-small cell lung cancer with multiple brain metastases may represent an overall safe and effective procedure (26). Ren et al. have reported a case of angiosarcoma of the head and face treated with anlotinib as first-line therapy, and the lesions were gradually controlled and evaluated as PR with a progression-free survival (PFS) of 10 months (12).

The case concerns an elderly female patient with postoperative recurrence of high-grade soft tissue sarcoma of the scalp who underwent surgery for a meningioma in 2006, followed by postoperative radiation therapy. Although the specific dose was unknown, given the 19-year latency period, sarcoma recurrence restricted to the prior surgical and radiation fields, and established radiation-associated sarcoma (RAS) risk from historical 50–60 Gy cranial irradiation, this lesion is highly likely to represent a RAS. Chemotherapy is the main first-line treatment option for advanced STS recommended by clinical guidelines (27). In several studies, treatment regimens based on doxorubicin are regarded as the gold standard for STS (28, 29). Yet, this patient was 77 years old, had experienced a recurrence following surgery, and had a history of coronary heart disease. Doxorubicin treatment carries a range of side effects, including cardiac and hematological reactions. In addition, the patient and her family refused chemotherapy, and consequently anlotinib was selected as the first-line treatment option. Here, we report for the first time an individualized treatment experience consisting of a combination of local and systemic therapy, including simultaneously integrated boost helical tomotherapy and anti-angiogenic targeted therapy with anlotinib as first-line long-term maintenance. The clinical outcomes demonstrated that the scalp lesion was gradually controlled and evaluated as CR. The clinical efficacy was long-lasting, and the patient maintained a good quality of life during this period until her death from a lung infection complicated by heart failure on 4 May 2026, with an OS of 11 months. This case proved to some extent that radiotherapy combined with anlotinib can be an effective treatment for high-grade scalp sarcoma. The limitation of our study is that this is only one case report, and further research with larger-scale clinical trials is required to validate our findings.

## Conclusion

Radiotherapy combined with anti-angiogenic targeted therapy may be of great clinical value for the treatment of HNSTS, and to some extent, may offer potential references for subsequent clinical decision-making. However, its long-term efficacy needs to be further studied.

## Data Availability

The original contributions presented in the study are included in the article/supplementary material. Further inquiries can be directed to the corresponding author.
